# Nectar traits differ between pollination syndromes in Balsaminaceae

**DOI:** 10.1093/aob/mcz072

**Published:** 2019-05-23

**Authors:** F Vandelook, S B Janssens, P Gijbels, E Fischer, W Van den Ende, O Honnay, S Abrahamczyk

**Affiliations:** 1 Meise Botanic Garden, Meise, Belgium; 2 Laboratory for Plant Conservation and Population Biology, Katholieke Universiteit Leuven, Leuven, Belgium; 3 Institut für Integrierte Naturwissenschaften – Biologie, Universität Koblenz-Landau, Koblenz, Germany; 4 Laboratory for Molecular Plant Biology, Katholieke Universiteit Leuven, Leuven, Belgium; 5 Nees-Institute for Biodiversity of Plants, University of Bonn, Bonn, Germany

**Keywords:** Adaptation, amino acids, Balsaminaceae, *Impatiens*, nectar, sugar

## Abstract

**Background and Aims:**

The attractiveness of nectar rewards depends both on the quantity of nectar produced and on its chemical composition. It is known that nectar quantity and chemical composition can differ in plant species depending on the main pollinator associated with the species. The main aims of this study were to test formally whether nectar traits are adapted to pollination syndromes in the speciose Balsaminaceae and, if so, whether a combination of nectar traits mirrors pollination syndromes.

**Methods:**

Comparative methods based on Ornstein–Uhlenbeck models were used to test whether nectar volume, nectar sucrose proportion, sugar and amino acid concentration and amino acid composition had evolved as a function of pollination syndromes in 57 species of Balsaminaceae. Cluster analysis and ordination were performed to derive clusters of species resembling each other in nectar composition.

**Key Results:**

Evolutionary models for nectar volume and nectar sucrose proportion performed best when including information on pollination syndrome, while including such information improve model fit neither for sugar and amino acid concentration nor for amino acid composition. A significant relationship emerged between pollination syndrome and the combined nectar traits.

**Conclusions:**

Our results show that nectar volume and nectar sucrose proportion evolve rapidly towards optimal values associated with different pollination syndromes. The detection of a signal indicating that nectar traits in combination are to a certain extent able to predict pollination syndromes in Balsaminaceae suggests that a holistic approach including the whole set of nectar traits helps us to better understand evolution of nectar composition in response to pollinators.

## INTRODUCTION

Nectar is considered the most common floral reward (Simpson and Neff, 1981), but it comes with a cost. Nectar quantity should be abundant enough to attract effective pollinators at a sufficient rate but small enough to force pollinators to visit various plant individuals, and to keep energy investment to a minimum (e.g. Pyke, 1984, 1991). The available nectar volume per flower is expected to depend on the energy requirement and nectar consumption mode of the most effective pollinators (Heinrich and Raven, 1972; Kevan and Baker, 1983). Flowers visited by large animals such as birds or bats usually produce more nectar than flowers that are mainly visited by smaller insects (Baker and Baker, 1983; Johnson and Nicolson, 2008). Associations between nectar quantity and pollinators are also assumed to be mediated by flower size: flowers with longer corolla tubes or flower spurs are commonly expected to have higher nectar volume due to mere allometry. Such a positive correlation has been found in some plant groups (Stanton and Young, 1994; Ornelas *et al.*, 2007; Tavares *et al.*, 2016), but was missing in others (Klinkhamer and van der Veen-van Wijk, 1999).

The attractiveness of nectar rewards depends not only on the quantity of nectar produced, but also on its chemical composition. A trade-off exists between energy content of the nectar and viscosity of the solution, which increase with increasing sugar concentration (Nicolson, 2011). In order to attract pollinators, nectar sugar concentration must be optimized to the pollinators’ energy requirements. Variation in nectar concentration between species has been associated with mouthpart structures and the type of feeding of the main pollinator groups (Kim *et al.*, 2011). Biophysical models showed that the optimal concentration for active or capillary suction feeders (birds, butterflies and moths) is 30–40 % while that for viscous dippers (bees and flies) is 50–60 % (Roubik and Buchmann, 1984; Kim *et al.*, 2011). In addition, selection may work not only on nectar concentration itself, but rather on corolla structure, with nectar in open flowers evaporating and thus becoming viscous more quickly, thereby influencing the rate of secretion and the chemical composition (Búrquez and Corbet, 1998).

Three main sugars are found in nectar, the disaccharide sucrose and its component monosaccharides, fructose and glucose. Within species, the proportions of the individual nectar sugars remain remarkably constant (Baker and Baker, 1982). Nectar sugar composition is related to the preferences of the main pollinator groups that visit the flowers as well as to flower characteristics. In this regard, several studies observed significantly higher nectar sucrose proportions in morphologically complex flowers frequented by specialized pollinator groups (hummingbirds, sunbirds, butterflies, moths or bees) as compared with plants with open flowers, pollinated by generalist pollinators such as most flies or birds feeding on nectar just occasionally (e.g. Percival, 1961; Baker and Baker, 1982; Gottsberger *et al.*, 1984; Abrahamczyk *et al.*, 2017*a*). However, few differences in nectar sucrose proportion were found between plants pollinated by different specialist pollinator groups, since all of them prefer a similar nectar sugar composition (Abrahamczyk *et al.*, 2017*a*). As a result, it is generally accepted that switches between pollinator groups are mostly related to changes in flower morphology and other nectar traits, such as volume or sugar concentration (Perret *et al.*, 2011).

Nectar is also a potential source of amino acids for pollinators (Baker and Baker, 1973). There has been debate, however, about whether amino acid concentration and composition in nectar are adapted to pollinators visiting the flowers (Gottsberger *et al.*, 1984; Baker and Baker, 1986). Commonly, the concentration of amino acids is larger if nectar is the main source of protein-building material for the pollinators (Kevan and Baker, 1983). This may explain why flowers visited by bees or birds, with pollen and insects, respectively, as alternative protein sources, often have lower amino acid concentrations in nectar as compared with flowers visited by butterflies (Baker and Baker, 1973; Kevan and Baker, 1983). All 20 protein-building amino acids are known to occur in nectars, but never in an equal amount (Kevan and Baker, 1983). Some amino acids such as alanine, arginine, serine, proline and glycine are almost always available, while others such as histidine and methionine are rarer (Haydak, 1970). The evolutionary significance of amino acid composition as gustatory stimulants or taste modifiers has been proposed (Chippendale, 1978), yet was never formally tested.

Balsaminaceae is a speciose family that contains >1000 species. Each year, several new species are described (e.g. Janssens *et al.*, 2009*b*, 2010, 2011, 2018; Abrahamczyk *et al.*, 2016; Fischer *et al.*, 2017). Its representatives are mainly growing in montane forests of the Old World tropics and sub-tropics (Yuan *et al.*, 2004; Janssens *et al.*, 2006). Despite its large number of species, the family consists of only two genera: *Impatiens* and *Hydrocera*, which split from each other in the early Miocene (Janssens *et al.*, 2009*a*). From a floral morphological point of view, Balsaminaceae are exceptionally diverse, representing various flower architectures and coloration patterns ranging from strikingly red or pink flowers with a spurred sepal of different sizes and shapes to small white flowers with an inconspicuous spur, or even tiny green-brownish cup-shaped, spur-less flowers (Yuan *et al.*, 2004; Abrahamczyk *et al.*, 2017*b*). However, all Balsaminaceae flowers are derived from a spurred, pentamerous ancestor in which a protruding spur was formed as an extrusion of the lower sepal containing the nectary (Grey-Wilson, 1980; Janssens *et al.*, 2012). Shape and size of the spur are interpreted as adaptations to pollinators but also as an excluding mechanism against nectar thieves (Grey-Wilson, 1980).

A large variety of pollinator groups has been documented for Balsaminaceae: individual species are pollinated by sunbirds, butterflies, moths, bees or flies (summarized in Abrahamczyk *et al.*, 2017*b*). However, most Balsaminaceae species lack detailed pollinator information. Therefore, the concept of pollination syndromes has successfully been applied to this family (Abrahamczyk *et al.*, 2017*b*). As such, Balsaminaceae can be regarded as an excellent study group to tackle questions about the evolution of nectar traits (Grey-Wilson, 1980; Ruchisansakun *et al.*, 2016; Abrahamczyk *et al.*, 2017*b*). Little is known on the nectar chemistry of Balsaminaceae. Nectar sugar concentration is a highly variable trait, and nectar mainly contains sucrose (Abrahamczyk *et al.*, 2017*a*). Nectar in Balsaminaceae also seems to have a species-specific amino acid composition (Rust, 1977).

In this study on the relationship between nectar traits and pollination syndromes in Balsaminaceae, the following hypotheses were tested: (1) nectar traits are adapted to pollination syndromes and thus we expect that evolutionary optimal values of individual traits differ between plant species with different pollination syndromes; (2) nectar volume and concentration are correlated to spur length in Balsaminaceae; and (3) all together we expect that nectar traits mirror pollination syndromes. Therefore, species pollinated by the same pollination syndrome show a composition of nectar traits typical for that pollination syndrome.

## MATERIALS AND METHODS

### Taxon sampling and experimental design

In total, 57 Balsaminaceae species were included in our analysis, representing the whole taxonomic and geographic diversity of the family, as well as all known pollination syndromes (Supplementary data Table S1). Per species we used one genetic individual or clone. Nectar collection was conducted in the pollinator-proof cabins of Bonn University Botanical Gardens. Since water availability can influence nectar volume and sugar concentration, we made sure that plants were always watered sufficiently. Furthermore, our results match the results of studies that measured nectar traits under natural conditions (Tian *et al.*, 2004; Bartoš *et al.*, 2012). Voucher specimens are deposited in the herbarium of Bonn. For genetic analyses, we expanded the phylogenetic data set of Janssens *et al.* (2009*a*) and Lozada-Gobilard *et al.* (2019) (Supplementary data Table S1). Since several newly added taxa are currently in the process of being officially described, they are here indicated with informal names. Based on the studies of Janssens *et al.* (2006, 2007, 2008), *Hydrocera triflora* was used as outgroup.

### Sampling and analysis of nectar

Per species, nectar was sampled from three freshly opened flowers using microcapillary tubes (Hirschmann Laborgeräte, Eberstadt, Germany). Nectar volume was determined by measuring the fluid column in the tubes. Nectar amino acid and sugar composition of all samples were analysed with high-performance anion exchange chromatography with pulsed amperometric detection analysis on an ICS3000 chromatography system (Thermo Scientific, Dionex, Sunnyvale, CA, USA). Nectar analysis was performed at a temperature of 32 °C, while the flow rate was 250 μL min^–1^.

For sugar composition analyses, a 15 μL diluted sample was analysed with both an analytical and a Guard CarboPac PA 100 column (2 × 250 mm and 2 × 50 mm, respectively; Dionex). Sugars were eluted in 90 mm NaOH and samples were run over an increasing NaAc gradient of 0 to 10 mm (6 min), 10 to 100 mm (10 min) and 100 to 175 mm (10 min). Before a new analysis started, columns were regenerated with 500 mm NaAc (1 min) and equilibrated with 90 mm NaOH (9 min).

For amino acid analyses, 15 μL of diluted sample was analysed with an AminoPac PA 10 column (2 × 50 mm; Dionex) in series with an analytical AminoPac PA 10 column (2 × 250 mm; Dionex). Amino acids were eluted in 50 mm NaOH for 14 min. Subsequent to elution, an increase of NaOH concentration from 50 to 80 mm (4 min) preceded an NaOH decrease from 80 to 60 mm (8 min). Simultaneously with the decrease in NaOH, the NaAc concentration increased from 0 to 400 mm. The final concentrations of 60 mm NaOH and 400 mm NaAc remained constant for 16 min. Before a new analysis started, columns were regenerated with 125 mm NaOH and 500 mm NaAc (1 min) and equilibrated with 50 mm NaOH (10 min).

The concentrations of the different sugars (fructose, glucose and sucrose) and amino acids in each sample were estimated by comparing the area under the chromatogram peaks with standards using Chromeleon software (Dionex). Total sugar and amino acid concentrations were estimated by calculating and summing the quantity of the different sugars and amino acids, and determining the concentration in relation to the total nectar volume. The replications yielded differences of only 5 %.

### Phylogenetic tree

Previous Balsaminaceae data sets of Janssens *et al.* (2009*a*) and Lozada-Gobilard *et al.* (2019) were extended with 13 new accessions (Supplementary data Table S1) to construct a phylogeny with a total of 57 taxa. Some taxa are currently in the process of officially being described and are therefore indicated with informal names. *Hydrocera triflora* was used as outgroup.

Total genomic DNA was isolated from silica-dried leaf material using a modified cetyltrimethylammonium bromide (CTAB) protocol (Doyle and Doyle, 1987), which is optimized for *Impatiens* by Janssens *et al.* (2006, 2008). The two nuclear *AP3*/*DEF* homologues (*ImpDEF1* and *ImpDEF2*) and the plastid *atpB–rbcL* intergenic spacer were amplified following Janssens *et al.* (2006, 2007). PCRs for all three gene markers investigated in this study consisted of 2 min initial denaturation at 94 °C and 30 cycles of 30 s denaturation at 94 °C, 30 s primer annealing at primer-specific temperature and 1 min extension at 72 °C. Primer annealing for *ImpDEF1*, *ImpDEF2* and *atpB–rbcL* was at 57, 55.5 and 51 °C, respectively. Amplification reactions were carried out on a GeneAmp PCR system 9700 (Applied Biosystems). Purified amplification products were sent to Macrogen, Inc. (Seoul, South Korea) for sequencing. Sequences obtained in this study were deposited at GenBank (Supplementary data Table S1).

Contiguous sequences were assembled using Geneious v7.0.6 (Biomatters, Auckland, New Zealand). Automatic alignments were carried out with MAFFT (Katoh *et al.*, 2002) under an E-INS-i algorithm, a 100PAM/*k* = 2 scoring matrix, a gap open penalty of 1.3 and an offset value of 0.123. Subsequent manual fine tuning of the aligned data set was done in Geneious v7.0.6. Congruency between the nuclear and chloroplast data sets was inferred by a partition homogeneity test as implemented in PAUP*4.0b10a (Swofford, 2003). The best-fit nucleotide substitution model for each plastid and nuclear data set was determined using jModelTest 2.1.4 (Posada, 2008) under the Akaike information criterion (AIC). The GTR + I + G model was found as the best fit for *ImpDEF1*, whereas the GTR + G model was calculated as the best substitution model for *ImpDEF2* and *atpB–rbcL*. A mixed-model approach was used in which the combined data set is partitioned in order to apply a different model of evolution on each DNA region (Ronquist and Huelsenbeck, 2003). Bayesian inference analyses were conducted with MrBayes v3.1 (Huelsenbeck and Ronquist, 2001) on three individual data partitions and a combined data matrix. Each analysis was run twice for 10 million generations. Trees were sampled every 2500 generations. Bayesian inference posterior probability (BPP) values between 0.50 and 0.95 as summarized in the 50 % majority-rule consensus tree are considered to be weakly supported, whereas only BPP values ≥0.95 are taken into consideration (Suzuki *et al.*, 2002). An ultrametric tree for further analyses was obtained using the BEAST 1.8.0 software package. BEAUti 1.10 was used to produce the input file for BEAST (Drummond and Rambaut, 2007). An unlinked, partitioned Bayesian Markov chain Monte Carlo (MCMC) analysis was conducted under a Yule speciation model and a relaxed lognormal clock. In total, 50 million generations were run with tree sampling at each 5000th generation. Chain convergence and explained sum of squares (ESS) parameter evaluation (ESS >200) was carried out with TRACER 1.6 (Rambaut *et al.*, 2014). TreeAnnotator 1.10 was used to compute the maximum clade credibility tree (Drummond and Rambaut, 2007). The age for *Impatiens* estimated by Janssens *et al.* (2009a) was chosen as calibration (normal distribution – mean value of 22.0 Ma – s.d. of 0.5).

### Nectar composition and spur length

All species were classified in one of five pollinator syndrome classes: (1) birds; (2) flies; (3) butterflies; (4); bees; and (5) both butterflies and bees, based on Abrahamczyk *et al.* (2017*b*). Length of the spur and length of the spur plus length of the spur carrying sepal (hereafter total spur length) were measured for a sub-set of 48 species (Supplementary data Table S1). The length of the spur-carrying sepal was measured from the tip of the opening to the point where the narrow part of the spur starts. This point where the spur starts until the tip of the spur was measured as spur length. All analyses involving flower morphological traits were performed on this sub-set of 48 species. Total nectar volume (μL), sugar percentage (% w/w), amino acid concentration (mm), nectar sucrose proportion as well as the fractions of 22 amino acids in the total amino acid content were determined. The amino acid composition was reduced into three variables by performing a principal component analysis (PCA) on the logit- (*x* + 0.01) transformed percentage of the 22 amino acids measured. The first two axes explained 51.02 % of the total variance, while a third additional axis explained only an additional 10.29 % of total variance. Correlations of the individual amino acids with the PCA axes are shown in Supplementary data Table S2. PCA was performed using the Vegan package (Oksanen, 2015) R version 3.1.1. (R Development Core Team, 2014).

### Trait evolution models

Multiple evolutionary models were tested for the different components of nectar composition and flower morphological traits. In the simplest scenarios, we tested whether nectar composition evolved as in a star-like phylogeny (no phylogenetic signal) or according to a pure drift Brownian motion model (phylogenetic signal *λ* = 1; Lynch, 1991; Pagel, 1999). Secondly, we ran models where the phylogenetic signal *λ* was estimated using a maximum likelihood (ML) procedure, where *λ* was constrained to vary between 0 and 1. Finally, a series of more complex Ornstein–Uhlenbeck (OU) models were tested. An OU model is a simple linear model that allows the quantification of the effects of both natural selection and inertia (Hansen, 1997; Butler and King, 2004; Hansen *et al.*, 2008). In its simplest form, the model assumes that the trait evolves towards a single hypothetical optimal trait value *θ*s. The model also includes both a parameter *α*, measuring the rate of adaptation towards the optimum, and a stochasticity component *σ*, which is a measure of the intensity of the random fluctuations in the evolutionary process. If *α* is large, species will adapt very rapidly to new conditions, whereas a low *α* makes ancient adaptations relatively more important (Hansen, 1997). We calculated a more intuitive measure of the phylogenetic signal in this OU model: phylogenetic half-life, *t*_1/2_ = log_e_(2)/*α*. This half-life indicates how long it takes before adaptation to a new selective regime is expected to be more influential than the constraints from the ancestral state (Hansen, 1997).

As the evolutionary optimum is expected to differ according to pollinator, we further refined this model so that it included different evolutionary optima based on different hypotheses regarding evolutionary adaptation to five different pollination syndromes (*θ*_1_ = birds, *θ*_2_ = butterflies, *θ*_3_ = bees, *θ*_4_ = flies and *θ*_5_ = butterflies and bees). Models of evolutionary adaptation to pollinator were based on an ML analysis of the ancestral trait states performed in R using the ace function in the APE package (Paradis, 2006). All nodes were assigned to the most likely pollination syndrome. The performance of the models was compared by means of the AICc (Akaike, 1973). Akaike weights were calculated following Wagenmakers and Farrell (2004). Nectar volume and amino acid content were log transformed, while sugar concentration, nectar sucrose proportion and amino acid percentage were logit [or logit (*x* + 0.001) in the case of zero values] transformed prior to analysis. Data shown are all back-transformed.

A cluster analysis and PCA were performed including all nectar chemical composition variables measured to visualize whether nectar chemical composition in general is related to pollination syndrome. For the cluster analysis, one Euclidean distance matrix was generated with standardized variables, which was then used to conduct a cluster analysis applying the UPGMA algorithm (unweighted pair group method with arithmetic means). The phylogenetic signal *λ* of pollination syndrome based on the cluster analyses was calculated to test whether species with a more similar nectar composition were more likely to be pollinated by the same pollination syndrome. This model was compared with a white noise model representing a star-like phylogeny using a likelihood ratio test. Alternatively, we correlated the Euclidean distance matrix of nectar chemical composition with a distance matrix of pollination syndrome using a Mantel test (*n* = 9999). The distance matrix of pollination syndrome was constructed manually, with the distance between different pollination syndrome equal to 1 and the distance between either bee- or butterfly-pollinated species and species pollinated by both bees and butterflies equal to 0.5. A PCA was performed including all (transformed) nectar traits measured in the study. In the case of the amino acid composition, we did not use each amino acid separately; instead, we used the values summarized in the three first PCA axes calculated before. All variables were scaled to unit variance before analysis. The performance of the resulting summarizing axes in different evolutionary models was analysed as described before for the individual variables. Analyses were performed using the Ade4 (Dray and Dufour, 2007), Geiger (Harmon *et al.*, 2009) and OUwie (Beaulieu and O’Meara, 2014) packages in R.

### Multiple regression models

The evolution of nectar volume as a function of pollination syndrome may very well depend on flower architecture. Therefore, we performed a phylogenetic regression of log nectar volume as a function of pollination syndrome and included spur length as a covariable. The phylogenetic regression consisted of an ML procedure, whereby the phylogenetic signal and regression model were estimated simultaneously. As a measure of the phylogenetic signal, we applied Pagel’s *λ* (Pagel, 1999; Revell, 2010). Analyses were performed using the APE (Paradis, 2006) and nlme (Pinheiro *et al.*, 2018) packages in R. Multiple regression methods based on the OU)model have been developed by Hansen *et al.* (2008) and Bartoszek *et al.* (2012). In these models, it is assumed that the response variable evolves toward a randomly changing optimum as a function of a predictor variable that has evolved according to a Brownian motion model of trait evolution (Hansen *et al.*, 2008). We analysed all regressions mentioned in the Results under this assumption of a changing optimum using the slouch package in R (Kopperud *et al.*, 2019). However, all regression models, except the relationship between amino acid concentration and amino acid composition, performed less well than non-phylogenetic models and therefore the results are not shown.

## RESULTS

### Nectar volume

**Fig. 1.**
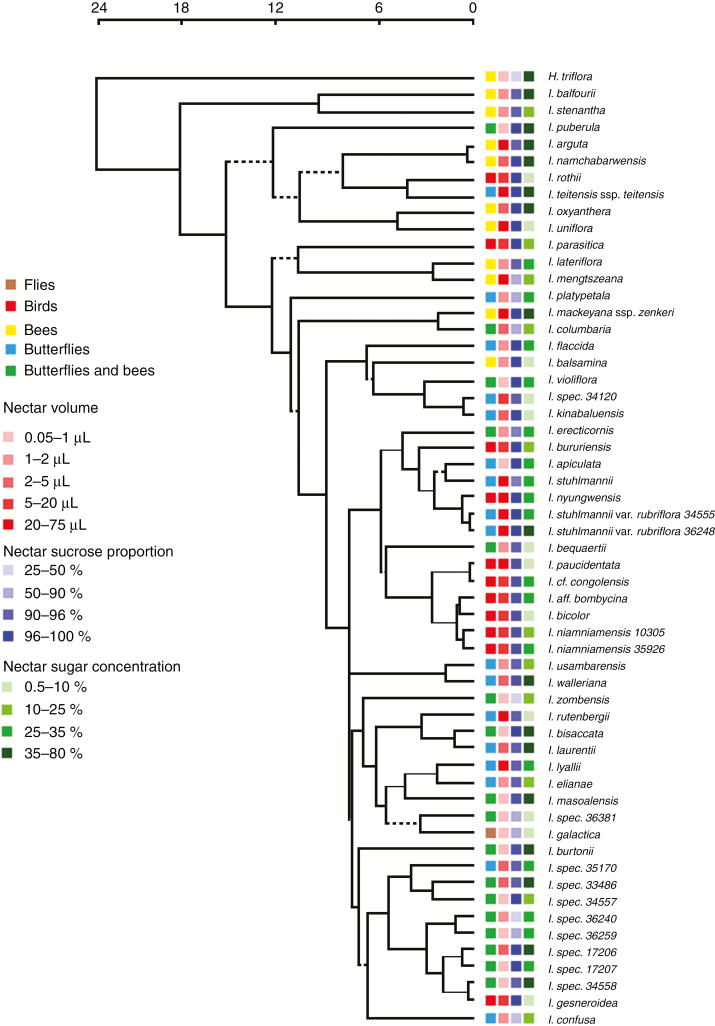
BEAST chronogram of the genus *Impatiens*. Dashed branches indicate lack of support by Bayesian analysis, thin branches show low support between 0.50 and 0.95, and thick branches indicate support above 0.95. Pollination syndrome and nectar volume, nectar sucrose proportion and nectar sugar concentration within the indicated ranges associated with each accession are indicated. Scale bar in Mya.

**Fig. 2.**
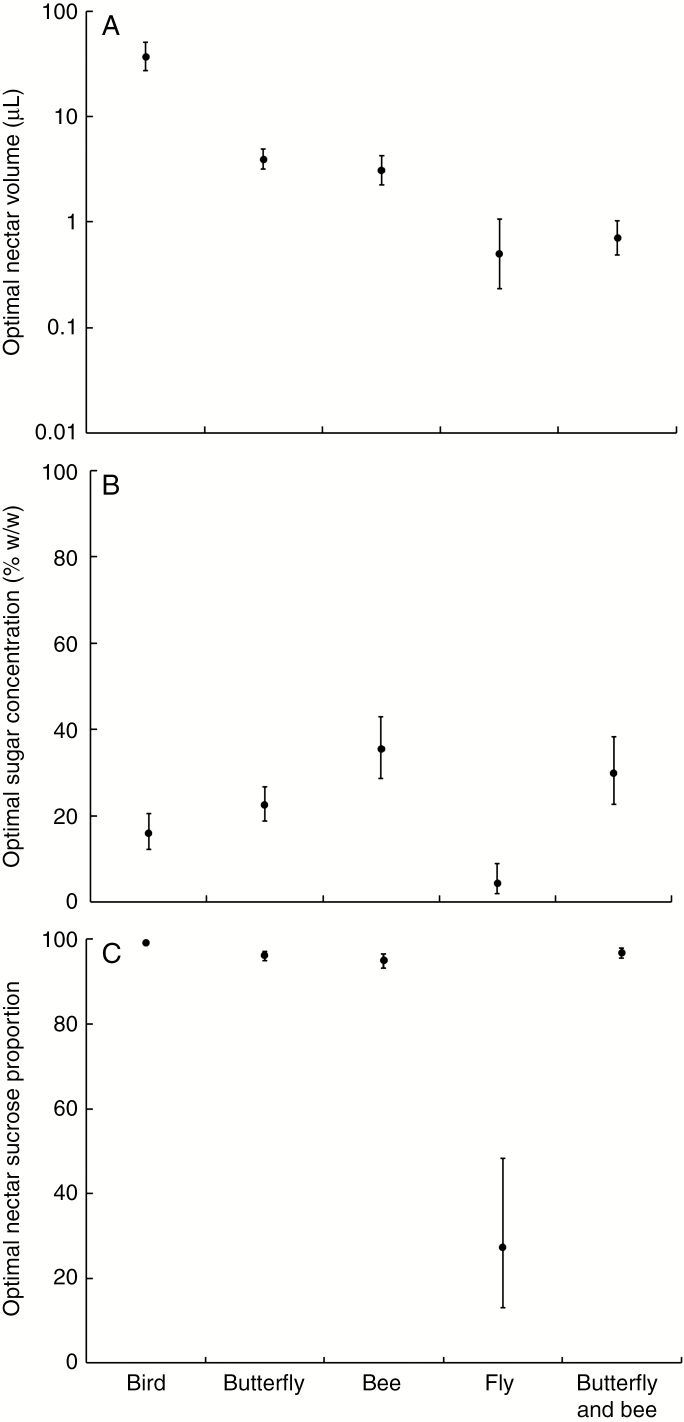
Evolutionary optimal trait values for (A) nectar volume, (B) sugar concentration and (C) nectar sucrose proportion (NSP) for species with different pollination syndromes. Optima in function of pollinator syndrome are based on Ornstein–Uhlenbeck models of trait evolution. These models represent the most likely evolutionary scenario for the respective traits. Error bars denote the s.e.

Nectar volume ranged between 72.16 μL in *Impatiens parasitica* and 0.06 μL in *Impatiens violiflora*. Significant phylogenetic signal, *λ* = 0.62, 95 % confidence interval (CI) 0.33–1.18, was observed (Fig. 1; Supplementary data Table S3) and the evolutionary model explaining distribution of nectar volume best was an OU model where nectar volume evolves toward optimal values as a function of pollination syndrome (Table 1; Supplementary data Table S4). The evolutionary optimal nectar volume was highest for bird-pollinated species (*θ* = 37.1, 95 % CI 27.6–50.0 μL) and lowest for fly-pollinated species (*θ* = 0.5, 95b % CI 0.2–1.0 μL; Fig. 2). All pollination syndromes (except bees vs. butterflies) showed non-overlapping evolutionary optimal nectar volumes (Fig. 2A). The phylogenetic half-life *t*_1/2_ (0.047 Ma) for the model with optima as a function of pollination syndrome is very short as compared with the total tree length (23.7 Ma), suggesting a strong pull towards the respective optima (Table 2).

**Table 1.** AICc values and Akaike weights [*w*_i_(AICc)] for evolutionary models on nectar components

	White	OU.s	OU.poll	BM.s	BM.rate	Lambda
Nectar volume	128.4	126.1	**80.6**	155.2	155.4	126.2
*w*_i_(AICc)	<0.01	<0.01	**1.00**	<0.01	<0.01	<0.01
Sugar concentration	**176.0**	178.3	177.0	215.6	194.0	178.3
*w*_i_(AICc)	**0.45**	0.14	0.27	<0.01	<0.01	0.14
Amino acid concentration	108.0	**101.0**	103.5	113.6	128.5	107.0
*w*_i_(AICc)	0.02	**0.73**	0.21	<0.01	<0.01	0.04
Nectar sucrose proportion	221.9	223.7	**215.9**	248.2	261.7	222.3
*w*_i_(AICc)	0.05	0.02	**0.90**	<0.01	<0.01	0.04
Amino acid PCA axis 1	**181.0**	183.2	190.2	236.8	227.4	183.2
*w*_i_(AICc)	**0.60**	0.20	<0.01	<0.01	<0.01	0.20
Amino acid PCA axis 2	**181.0**	183.7	192.1	238.6	222.2	183.2
*w*_i_(AICc)	**0.62**	0.16	<0.01	<0.01	<0.01	0.21
Amino acid PCA axis 3	**181.0**	183.7	185.3	244.8	220.8	183.2
*w*_i_(AICc)	**0.59**	0.15	0.07	<0.01	<0.01	0.20
Nectar PCA axis 1	105.7	**101.7**	106.0	118.2	134.1	107.9
*w*_i_(AICc)	0.10	**0.77**	0.09	<0.01	<0.01	0.04
Nectar PCA axis 2	105.7	105.0	**87.1**	137.6	137.7	107.0
*w*_i_(AICc)	<0.01	<0.01	**1**	<0.01	<0.01	<0.01
Nectar PCA axis 3	**105.7**	108.9	112.3	164.8	133.8	107.9
*w*_i_(AICc)	**0.64**	0.13	0.02	<0.01	<0.01	0.21

The lowest AICc values indicating the best model fit are given in bold. Akaike weight can be interpreted as the probability that the model with the lowest AICc is the best model.

White, non-phylogenetic model; OU.s, Ornstein–Uhlenbeck model with single evolutionary optimum; OU.poll, Ornstein–Uhlenbeck model evolutionary optima in function of pollination syndrome; BM.s, Brownian motion model with constant evolutionary rate; BM.rate, Brownian motion with different rate parameters; Lambda, model the including phylogenetic signal *λ* estimated using a maximum likelihood procedure.

**Table 2.** Parameter estimates for OU models of nectar volume, sugar concentration and nectar sucrose proportion with adaptive optima as a function of pollination syndrome or, in the case of amino acid concentration and PCA axis 3, as a function of a single optimum value

	*σ* ^2^	α	*t*_1/2_ (Ma)
Nectar volume	5.42	14.9	0.047
Sugar concentration	29.4	15.0	0.046
Nectar sucrose proportion	58.1	14.9	0.047
Amino acid concentration	0.26	0.45	1.540
PCA axis 3	7.86	3.46	0.200

*σ* ^2^, magnitude of stochasticity component; *α*, rate of adaptation; *t*_1/2_ phylogenetic half-life.

Full model parameters are given in Supplementary data Table S4.

A significant positive relationship exists between total spur length and nectar volume (coefficient = 1.85 ± 0.38; *t* = 4.92; *P* < 0.001; AICc = 85.5). This phylogenetic regression model between total spur length and nectar volume is, however, outperformed by a model that also includes pollination syndromes (Table 3; AICc = 59.6). Including phylogenetic information did not improve the model fit, as the lowest AICc was found at *λ* = 0 for both models.

**Table 3.** Phylogenetic generalized least squares model with log nectar volume as response variable, pollination syndrome as predictor variable and log total spur length as covariable

	Coefficient	s.e.	*t*-value	*P*-value
Intercept	1.97	0.25	7.76	<0.001
Log total spur length	0.99	0.48	2.01	0.04
Pollination syndrome				
Butterfly	–0.92	0.16	–5.66	<0.001
Bee	–0.85	0.21	–4.01	<0.001
Fly	–1.58	0.45	–3.50	0.001
Bee and butterfly	–1.22	0.25	–4.94	<0.001

Species pollinated by butterflies, bees, flies and both bee and butterflies are tested against bird pollinated flowers.

### Nectar sugar concentration

Sugar concentration in nectar varied between 76.9 % in *Impatiens balfourii* and 0.73 % in *Impatiens sp. nov. 36381.* The *λ* value for sugar concentration converged to 0, indicating that phylogenetic signal was absent (Supplementary data Table S3). Nonetheless, the best performing model for sugar concentration evolution was an OU model with optima as a function of pollination syndrome (Table 1; Supplementary data Table S4). Optimal nectar sugar concentrations between 22 and 36 % are found in flowers pollinated by butterflies, bees and both butterflies and bees (Fig. 2B), while lower concentrations of about 15 % were found in bird-pollinated flowers and of <5 % in fly-pollinated flowers. The phylogenetic half-life *t*_1/2_ (0.046 Ma) for the model of nectar sugar concentration with optima as a function of pollination syndrome was also very short as compared with the total tree length (Table 2). The stochasticity component (*σ*^2^), however, was higher as compared with that of nectar volume, indicating stronger random changes in nectar sugar concentration.

There was no significant correlation between nectar volume and sugar concentration (*t* = –1.52; P = 0.13; *λ* = 0). However, when excluding the fly-pollinated species, which has extremely low nectar volumes and sugar concentration, a significant negative correlation was found between nectar volume and sugar concentration (*t* = –2.33; *P* = 0.02; *λ* = 0). There was no significant correlation between sugar concentration and length of the spur (*t* = 1.61; *P* = 0.11; *λ* = 0; AICc = 138.2) or total spur length (*t* = –0.70; *P* = 0.49; *λ* = 0; AICc = 140.3). Including pollination syndrome in the model improved the model fit (AICc = 134.2 and AICc = 133.5, respectively), but still no significant correlations between sugar concentration and spur length or total spur length were detected (*P* = 0.42 and *P* = 0.27, respectively).

### Nectar sucrose proportion

Nectar sucrose proportion ranges from 99.8 % in *Impatiens bombycina* to 25.2 % in *Impatiens sp. nov. 36381*. The phylogenetic signal (*λ* = 0.65, 95 % CI 0.31–1.36) was high but not significantly different from zero (Table 1; Supplementary data Table S4). A high nectar sucrose proportion was, however, dominant in three clades, while a low nectar sucrose proportion can be found spread throughout the phylogeny (Fig. 2C). Again, the best model explaining evolution of nectar sucrose proportion is an OU model taking pollination syndrome into account (Table 1). Optimal nectar sucrose proportion was 99.1 % (95 % CI 89.6–99.4) in bird-pollinated Balsaminaceae species and showed no overlap with the optima for the insect-pollinated species (Fig. 2C). In the fly-pollinated species, the optimal nectar sucrose proportion was much lower (about 27 %), because all three sugars were present in a fairly equal amount (glucose 24 %, fructose 35 % and sucrose 27 %). Again, the phylogenetic half-life *t*_1/2_ (0.047 Ma) for the model of nectar sugar concentration with optima as a function of pollination syndrome was very short as compared with the total tree length, and the stochasticity component (*σ*^2^) was very high (Table 2).

There was no significant correlation between nectar sucrose proportion and nectar sugar concentration (*t* = 0.49; *P* = 0.62; *λ* = 0), but nectar sucrose proportion was significantly higher in species with larger nectar volumes (*t* = 2.60; *P* = 0.01; *λ* = 0). No significant correlation was found between total length of the spur and nectar sucrose proportion (*t* = 0.57; *P* = 0.57; *λ* = 0). The model fit was improved by including pollination syndrome in the model (AICc 172.0 vs. AICc 181.5 without pollination syndrome), but did not result in a significant correlation between spur length and nectar sucrose proportion (Table 4).

**Table 4.** Generalized least squares model with logit nectar sucrose proportion as response variable, pollination syndromes as predictor variable and log (total spur length) as covariable

	Coefficient	s.e.	*t*-value	*P*-value
Intercept	4.74	0.49	9.67	<0.001
Log total spur length	0.07	0.43	0.18	0.86
Pollination syndrome				
Butterfly	–1.63	0.53	–3.09	0.003
Bee	–0.98	0.67	–1.46	0.15
Fly	–5.48	1.43	–3.84	<0.001
Bee and butterfly	–1.30	0.74	–1.75	0.09

Species pollinated by butterflies, bees, flies and both bee and butterflies are tested against bird-pollinated flowers.

### Amino acid concentration

Amino acid concentration in the Balsaminaceae species studied varied between 0.20 and 69.70 mm. There was a marginally significant phylogenetic signal (*λ* = 0.54, 95 % CI 0.20–1.45) in amino acid concentration (Supplementary data Table S2), and the best model describing evolution of nectar concentration was an OU model with a single evolutionary optimum (Table 1; Supplementary data Table S4). The evolutionary optimal amino acid concentration was 3.40 mm (95 % CI 2.68–4.28). The phylogenetic half-life was high for amino acid concentration, while the stochasticity component was very low (Table 2), indicating slow changes in amino acid concentration in nectar of Balsaminaceae.

### Amino acid composition

Six amino acids (alanine, arginine, glycine, threonine, valine and lysine) occurred in all species studied, while methionine was found in only 17 % of the species studied (Supplementary data Table S5). The first PCA axis correlated most strongly with arginine, phenylalanine, ornithine and lysine, the second axis correlated strongly with isoleucine and glutamine, and the third axis correlated strongly with methionine and tyrosine (Supplementary data Table S2). Phylogenetic signal was absent (*λ* = 0) for all three PCA axes (Supplementary data Table S3). The models that explained the distribution of amino acid composition across the phylogeny best were those that did not take phylogeny into account (Table 1; Supplementary data Table S4). When looking at the amino acids separately, significant phylogenetic signal was only observed for tyrosine and methionine (Table Supplementary data S3), while high but not significant *λ* values were found for glutamine, histidine and valine. In general, the phylogenetic signal in amino acids was absent or weak. A significant negative correlation was found between amino acid concentration and PCA1 (*t* = –3.64; *P* < 0.001; *λ* = 0.75), PCA2 (*t* = 2.87; *P* = 0.01; *λ* = 0.87) and PCA3 (*t* = –2.46; *P* = 0.02; *λ* = 0.72).

### Cluster analysis and ordination

**Fig. 3.**
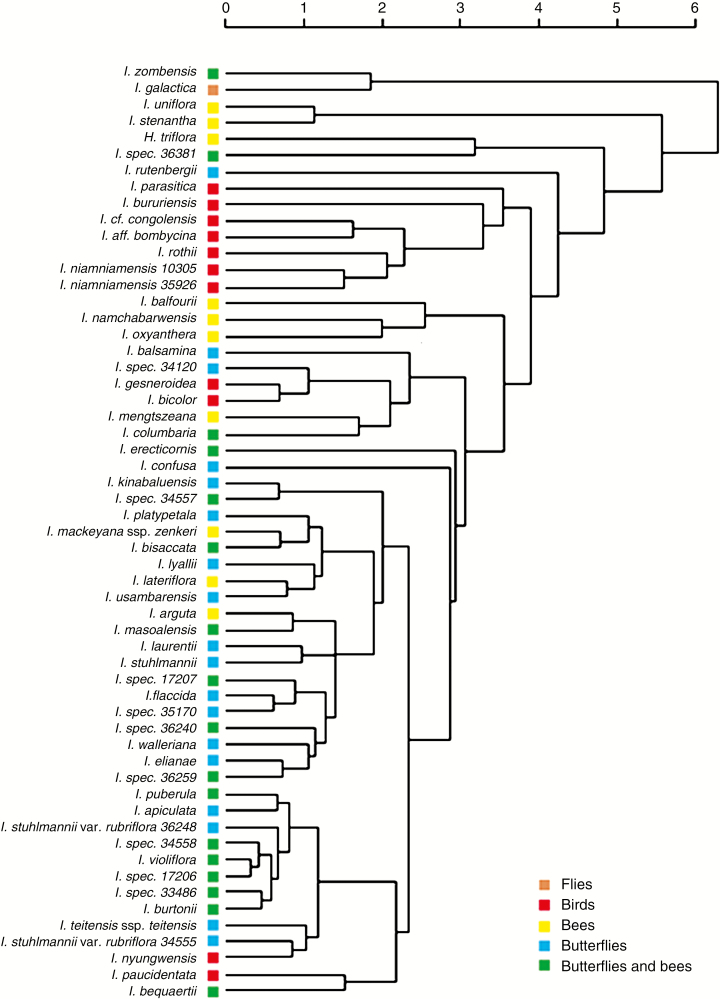
Cluster analysis generated from a Euclidean distance matrix based on standardized nectar chemical composition variables: volume, sucrose proportion, sugar concentration, amino acid concentration and three PCA axes summarizing amino acid composition. Pollination syndromes were plotted on the resulting tree.

Significant phylogenetic signal for pollination syndrome (likelihood ratio test *P* = 0.001; *λ* = 0.97) as a function of clustering of nectar chemical composition was detected (Fig. 3). A Mantel test also showed a significant correlation (*P* < 0.001) between pollination syndrome and the combined nectar traits. Two large groups could be discerned in Fig. 3. First, species pollinated by butterflies and by both butterflies and bees formed one cluster together with a few bee-pollinated species and two bird-pollinated species. Secondly, the majority of the bird- and bee-pollinated species grouped together with the fly-pollinated species.

The first axis of the PCA including all nectar variables explained about 32 % of the variation, while the second and third axes explained 20 and 15 %, respectively. A clear grouping pattern based on pollination syndrome was not apparent, apart from bird-pollinated species (Supplementary data Fig. S1). Evolutionary model comparisons using the PCA axes summarizing nectar composition showed that a non-phylogenetic model performed best for axis 3, while an OU model with a single evolutionary optimum performed best for axis 1 (Table 1). Interestingly, for axis 2, an OU model with optima as a function of pollination mode strongly outperformed all other models. The second PCA axis correlated significantly and negatively with nectar volume, nectar sucrose proportion and amino acid composition axis 2, and significantly positive with sugar concentration and amino acid composition axis 3 (Supplementary data Table S6).

## DISCUSSION

Our results showed that Balsaminaceae species with the same pollination syndrome tend to have similar nectar characteristics, whereas nectar traits between syndromes differ significantly. As we will outline below, there is strong evidence that the evolution of certain nectar characters has occurred as an adaptation to pollinator preferences. Our analyses indicate that especially nectar volume and sucrose proportion of Balsaminaceae nectar have evolved in close association with pollination syndromes, while there are indications that the same is true for sugar and amino acid concentration. The phylogenetic signal *λ* was either zero, which indicates no phylogenetic signal at all, or a value between zero and one, which indicates that adaptation may have occurred alongside drift, as was indeed shown by the fact that the OU models performed best for some variables. No evidence was found that amino acid composition in nectar evolved as an adaptation to pollination syndromes, maybe due to its sensitivity to environmental factors such as nutrient availability (Nicolson and Thornburg, 2007).

As commonly observed in other studies, bird-pollinated Balsaminaceae species produce rather large amounts of extremely sucrose-rich nectar, yet in low concentrations (Nicolson and Fleming, 2003; Goldblatt and Manning, 2005). This combination of unique traits suggests that stabilizing selection on nectar traits in bird-pollinated species is very strong. Instead, nectar traits of species pollinated by bees, butterflies or a combination of both are much more similar, having lower amounts of sucrose-rich, more concentrated nectar. In particular, the nectar of bee-pollinated Balsaminaceae species is characterized by higher amino acid concentrations, whereas lower volumes are typical for nectar of species pollinated by a combination of bees and butterflies. This high concentration of amino acids in nectar of bee-pollinated species is a little surprising since high amino acid concentrations in nectar are normally expected in plants pollinated by butterflies, for which nectar is the only source of food (Nicolson, 2007). The most aberrant nectar is produced by the only fly-pollinated species, with extremely low volume and sugar concentration but high contents of amino acids and hexoses, which is typical for fly-pollinated species (Gardener and Gillman, 2002; Nicolson, 2007; Abrahamczyk *et al.*, 2017*a*).

The relationship between spur length and nectar volume was overlaid by pollination syndrome, because, even after accounting for spur length, nectar volume was still significantly larger in bird-pollinated Balsaminaceae flowers compared with all other syndromes, even though butterfly-pollinated species have the longest spurs. This suggests that the relationship between total spur length and nectar volume is not a mere allometric one. A long, but partly empty spur may act much more as a morphological filter, allowing only morphologically adapted pollinators to reach the nectar. The same explanation might also be valid for the missing correlation between sugar concentrations and spur length in Balsaminaceae since only a correlation of these traits would explain the hypotheses that diluted nectar is a secondary consequence of deep tubular flowers, which minimize water evaporation (Plowright, 1987), or that species with especially long and narrow spurs, as commonly found in butterfly-pollinated flowers, have nectar with a low sugar concentration to compensate for increased adhesion by lower nectar viscosity.

An interesting result of our study was the detection of a signal indicating that nectar traits in combination are to a certain extent able to predict pollination syndromes in Balsaminaceae. In particular, bird-pollinated species are separated from the other pollination syndromes mainly due to a significantly higher nectar volume and extremely sucrose-rich nectar. This means that ‘nectar syndromes’, which are just one part of the classical pollination syndromes, contain sufficient information to predict pollination syndromes and are thus much more informative than their individual traits which are just able to differentiate between the most extreme states. It also means that nectar traits evolved in parallel to the fast evolving flower architecture in Balsaminaceae. Moreover, the rate parameters estimated in the evolutionary models indicate that nectar volume, nectar sucrose proportion and sugar concentration also evolve at a rapid rate and that there is a strong pull towards the optimal values associated with the respective pollination syndromes. These results are supported by a recent study on flower traits in relation to pollination syndrome in *Penstemon* (Katzer *et al.*, 2019).

Our results further underline the importance of generating quantitative data for predicting pollination syndromes (see discussion in Abrahamczyk *et al.*, 2017*b*) and document that nectar traits are as useful for predicting pollination syndromes as flower morphometrics. Since we only studied a small sub-set of Balsaminaceae species, we expect that the observed patterns will become even clearer when the study is expanded to include more species. Also, further studies in other plant families should show whether this pattern found in Balsaminaceae holds across the entire angiosperms.

## SUPPLEMENTARY DATA

Supplementary data are available online at https://academic.oup.com/aob and consist of the following. Figure S1: first two axes of a principal component analysis of all nectar traits studied of 57 Balsaminaceae species. Table S1: species and data used in the study. Table S2: *R* values for Pearson correlations of three PCA axes with amino acids. Table S3: phylogenetic signal *λ* of all nectar composition variables included in the study. Table S4: model parameters for evolutionary models on nectar components. Table S5: frequency of the different amino acids in 395 angiosperm species previously studied and in the 57 Balsaminaceae species and 15 bee-pollinated Balsaminaceae species from this study. Table S6: *R* values for Pearson correlations of three PCA axes with nectar traits.

mcz072_suppl_Supplementary_Figure-S1

mcz072_suppl_Supplementary-Data-Table-S1

mcz072_suppl_Supplementary-Data-Table-S2

mcz072_suppl_Supplementary-Data-Table-S3

mcz072_suppl_Supplementary-Data-Table-S4

mcz072_suppl_Supplementary-Data-Table-S5

mcz072_suppl_Supplementary-Data-Table-S6
