# Supplementary material for: Nectar traits differ between pollination syndromes in Balsaminaceae
Source: Ann Bot. 2019 May 23;124(2):269–79. doi: 10.1093/aob/mcz072 (PMC6758581; doi:10.1093/aob/mcz072)
Supplement: mcz072_suppl_Supplementary_Figure-S1 [file mcz072_suppl_supplementary_figure-s1.docx]

Fig. S1 First two axes of a principal component analysis of all nectar traits studied (volume, sugar concentration, amino acid concentration, amino acid composition and sucrose proportion) of 57 Balsaminaceae species. Dots were colored differently depending on pollination syndrome.
