# Supplementary material for: Nectar traits differ between pollination syndromes in Balsaminaceae
Source: Ann Bot. 2019 May 23;124(2):269–79. doi: 10.1093/aob/mcz072 (PMC6758581; doi:10.1093/aob/mcz072)
Supplement: mcz072_suppl_Supplementary-Data-Table-S2 [file mcz072_suppl_supplementary-data-table-s2.docx]

|  |  | | |
| --- | --- | --- | --- |
| Amino acid | Axis 1 | Axis 2 | Axis 3 |
| Arginine (%) | **0.89** | -0.23 | -0.12 |
| Ornithine (%) | **0.80** | -0.10 | -0.17 |
| Lysine (%) | **0.67** | **-0.34** | 0.04 |
| Glutamine (%) | -0.24 | **0.64** | -0.06 |
| Asparagine (%) | -0.01 | **0.33** | -0.02 |
| GABA (%) | **-0.51** | -0.04 | -0.31 |
| Beta-alanine (%) | **-0.33** | 0.11 | -0.01 |
| Alanine (%) | -0.19 | **0.44** | **0.51** |
| Threonine (%) | **-0.34** | -0.11 | 0.13 |
| Glycine (%) | **0.57** | 0.13 | -0.15 |
| Valine (%) | -0.14 | -0.24 | 0.10 |
| Serine (%) | **-0.64** | **-0.33** | -0.04 |
| Proline (%) | **-0.32** | -0.10 | -0.01 |
| Isoleucine (%) | 0.19 | **0.65** | 0.14 |
| Leucine (%) | -0.20 | 0.35 | **0.42** |
| Methionine (%) | -0.08 | 0.12 | **0.80** |
| Histidine (%) | **0.58** | 0.04 | 0.25 |
| Phenylalanine (%) | **0.75** | -0.20 | -0.01 |
| Glutamic acid (%) | -0.03 | **0.47** | **-0.46** |
| Aspartic acid (%) | 0.06 | 0.20 | **-0.49** |
| Cystine (%) | -0.03 | **0.54** | 0.03 |
| Tyrosine (%) | 0.08 | 0.12 | **0.84** |

Table S2. R values for Pearson correlations of three PCA axes with amino acids. Significant correlations (α < 0.05) in bold.
