# Supplementary material for: Nectar traits differ between pollination syndromes in Balsaminaceae
Source: Ann Bot. 2019 May 23;124(2):269–79. doi: 10.1093/aob/mcz072 (PMC6758581; doi:10.1093/aob/mcz072)
Supplement: mcz072_suppl_Supplementary-Data-Table-S3 [file mcz072_suppl_supplementary-data-table-s3.docx]

|  | Lambda | P-value |  | Lambda | P-value |
| --- | --- | --- | --- | --- | --- |
| Nectar volume (µL) | 0.62 | 0.04 | Threonine (%) | 0 | 1 |
| Sugar conc. (%) | 0 | 1 | Glycine (%) | 0 | 1 |
| Amino acid conc. (M) | 0.54 | 0.07 | Valine (%) | 0.45 | 0.59 |
| NSP | 0.65 | 0.17 | Serine (%) | 0 | 1 |
| AA PCA1 | 0 | 1 | Proline (%) | 0 | 1 |
| AA PCA2 | 0 | 1 | Isoleucine (%) | 0 | 1 |
| AA PCA3 | 0 | 1 | Leucine (%) | 0 | 1 |
| Arginine (%) | 0 | 1 | Methionine (%) | 0.95 | 0.02 |
| Ornithine (%) | 0 | 1 | Histidine (%) | 0.29 | 0.48 |
| Lysine (%) | 0 | 1 | Phenylalanine (%) | 0 | 1 |
| Glutamine (%) | 0.61 | 0.20 | Glutamic acid (%) | 0 | 1 |
| Asparagine (%) | 0 | 1 | Aspartic acid (%) | 0 | 1 |
| GABA (%) | 0 | 1 | Cystine (%) | 0 | 1 |
| Beta-alanine (%) | 0 | 1 | Tyrosine (%) | 0.90 | 0.01 |
| Alanine (%) | 0 | 1 |  |  |  |

Table S3. Phylogenetic signal lambda of all nectar composition variables included in the study. P-value (α = 0.05) based on the likelihood ratio test.
