# Supplementary material for: Nectar traits differ between pollination syndromes in Balsaminaceae
Source: Ann Bot. 2019 May 23;124(2):269–79. doi: 10.1093/aob/mcz072 (PMC6758581; doi:10.1093/aob/mcz072)
Supplement: mcz072_suppl_Supplementary-Data-Table-S5 [file mcz072_suppl_supplementary-data-table-s5.docx]

|  | Angiosperms | Balsaminaceae | Bee pollinated Balsaminaceae |
| --- | --- | --- | --- |
| Alanine | 96 | 100 | 100 |
| Arginine* | 90 | 100 | 100 |
| Serine+ | 89 | 48 | 60 |
| Proline+ | 87 | 48 | 40 |
| Glycine+ | 84 | 100 | 100 |
| Isoleucine* | 73 | 91 | 80 |
| Threonine* | 67 | 100 | 100 |
| Valine* | 66 | 100 | 100 |
| Leucine* | 65 | 36 | 47 |
| Glutamic acid | 62 | 69 | 67 |
| Cysteine, | 55 | 53 | 60 |
| Phenylalanine* | 55 | 98 | 93 |
| Tyrosine | 52 | 98 | 93 |
| Lysine* | 41 | 100 | 100 |
| Glutamine | 41 | 95 | 87 |
| Aspartic acid | 32 | 66 | 67 |
| Asparagine | 27 | 97 | 100 |
| Methionine* | 20 | 17 | 33 |
| Histidine* | 19 | 98 | 93 |
| Nonprotein | 36 | 56 | 57 |

Table S5. Frequency of the different amino acids in 395 angiosperm species studied by Baker and Baker (1973) and in the 57 Balsaminaceae species and 15 bee pollinated Balsaminaceae species from this study. * Amino acid are necessary for adult honeybees.
