# Supplementary material for: Nectar traits differ between pollination syndromes in Balsaminaceae
Source: Ann Bot. 2019 May 23;124(2):269–79. doi: 10.1093/aob/mcz072 (PMC6758581; doi:10.1093/aob/mcz072)
Supplement: mcz072_suppl_Supplementary-Data-Table-S6 [file mcz072_suppl_supplementary-data-table-s6.docx]

|  |  | | |
| --- | --- | --- | --- |
| Nectar composition | Axis 1 | Axis 2 | Axis 3 |
| Volume | 0.326 | **-0.744** | 0.303 |
| Sugar concentration | **0.612** | **0.425** | 0.075 |
| NSP | **0.669** | **-0.486** | -0.168 |
| AA concentration | **-0.808** | -0.060 | -0.045 |
| AA composition axis 1 | **0.707** | 0.026 | -0.092 |
| AA composition axis 2 | -0.231 | **-0.513** | **-0.738** |
| AA composition axis 3 | 0.284 | **0.433** | **-0.616** |

Table S6. R values for Pearson correlations of three PCA axes with amino acids. Significant correlations (α < 0.05) in bold.
